# Factors influencing the growth of preterm infants in the first two years of life

**DOI:** 10.1590/1984-0462/2025/43/2024297

**Published:** 2025-11-14

**Authors:** Milene de Moraes Sedrez Rover, Lígia Maria Suppo de Souza Rugolo, Cláudia Silveira Viera

**Affiliations:** a Universidade Estadual do Oeste do Paraná, Cascavel, PR, Brazil.; b Universidade Estadual Paulista Júlio de Mesquita Filho, São Paulo, SP, Brazil.

**Keywords:** Premature newborn, Growth, Risk factors, Self-efficacy, Recém-nascido prematuro, Crescimento, Fatores de risco, Autoeficácia

## Abstract

**Objective:**

To identify maternal and neonatal factors associated with growth outcomes in preterm infants (PT) born before 33 weeks of gestation, assessed at 24 months corrected age (CA).

**Methods:**

Prospective cohort study with PT <33 weeks’ gestation, discharged from the Neonatal Intensive Care Unit of a university hospital between 2019 and 2021, followed at the high-risk outpatient clinic. Variables analyzed: Maternal — sociodemographic aspects, gestational morbidities, selfefficacy score (confidence that parents have in their ability to perform the tasks of parenthood); Newborn — data from birth, hospital stay and post-discharge, measures of weight, height and head circumference at birth, discharge and follow up (seven evaluations). Outcome: Z scores of anthropometric measurements and growth failure (Z score <-2) at 24 months CA. Data were analyzed by hierarchical logistic or linear regression models in blocks.

**Results:**

99 PT with a mean gestational age of 30.2±2.0 weeks were studied. Intrauterine growth restriction (fetus that fails to reach its growth percentage, caused by maternal/placental factors) and being born small for gestational age (<10^th^ percentile according to Fenton’s calculator) were predictors of growth failure. Time to achieve full enteral feeding, necrotizing enterocolitis and maternal self-efficacy were associated with anthropometric measurements at 24 months.

**Conclusions:**

The growth of PT in the first 24 months CA is influenced by the nutritional condition at birth, nutritional evolution during hospitalization and maternal self-efficacy. Optimizing nutritional practices for PT and stimulating maternal self-efficacy are possibilities for improving growth in the early years.

## RESUMO

**Objetivo:**

Identificar fatores maternos e do recém-nascido que influenciam o crescimento de prematuros menores de 33 semanas aos 24 meses de idade corrigida (IC).

**Métodos:**

Coorte prospectiva de prematuros <33 semanas, egressos da Unidade de Terapia Intensiva Neonatal de hospital universitário entre 2019 e 2021, acompanhados no ambulatório de alto risco. Variáveis analisadas: Maternas — aspectos sociodemográficos, morbidades gestacionais, escore de autoeficácia (confiança dos pais na sua capacidade de desempenhar tarefas da parentalidade); Recém-nascido — dados do nascimento, evolução na internação e após a alta hospitalar, medidas de peso, estatura e perímetro cefálico ao nascimento, alta e follow-up (sete avaliações). Desfecho: escores Z das medidas antropométricas e falha no crescimento aos 24 meses de IC. Modelos de regressão logística ou linear hierárquica em blocos foram utilizados para testar associações entre crescimento e variáveis maternas e do recém-nascido.

**Resultados:**

Foram estudados 99 prematuros, com idade gestacional média de 30,2±2,0 semanas. Restrição do crescimento intrauterino (feto que não atinge seu percentual de crescimento por fatores maternos/placentários) e ser pequeno para idade gestacional (abaixo do percentil 10 segundo a curva de Fenton) foram preditores de falha no crescimento. O tempo para atingir dieta plena, a enterocolite necrosante e a autoeficácia materna influenciaram nas medidas antropométricas aos 24 meses.

**Conclusões:**

O crescimento de prematuros nos primeiros 24 meses de IC é influenciado pela condição nutricional ao nascimento, pela evolução nutricional na internação e pela autoeficácia materna. Otimizar as práticas nutricionais para prematuros e estimular a autoeficácia materna são possibilidades de melhorar o crescimento nos primeiros anos.

## INTRODUCTION

 Prematurity is the main cause of mortality in children under five years of age, and approximately 15% of the total annual 13.4 million preterm infants (PI) are born before 32 weeks of gestational age (GA) and require greater care due to the high risk of morbidity and sequelae in the short and long term.^1,2^

 There is great concern about the growth pattern of premature newborns because, although accelerated initial growth is beneficial for neurodevelopment, it can lead to future metabolic disorders.^3,4^ In premature infants born before 30 weeks, the rate of z score <-2 in relation to weight is 24% at 36 weeks of corrected age, decreasing progressively to 6% at 21 months and 5% at 36 months.^5^ Growth failure (Z score <-2) in the first years is frequent and influenced by several factors, including intrauterine growth restriction and postnatal growth restriction that is associated with the degree of prematurity, birth weight, nutritional practices, time to reach full feeds during Neonatal Intensive Care Unit (NICU) stay, hypothyroidism and weight for age Z-score at three months corrected age (CA), morbidities such as bronchopulmonary dysplasia, severe retinopathy of prematurity, ventilatory support and longer hospital stay.^6-9^ Most PI without major neonatal morbidities catch up to growth chart curves by three years CA.^5^

 Besides biological and sociodemographic factors, psychosocial aspects can influence weight gain and, consequently, growth.^3,10^ They include the number of pregnancies, births, live children, premature birth, and presence of maternal depression. The prevalence of postpartum depression in mothers of full-term newborns is 20.2%, increasing the risk in mothers of premature infants by 1.7 times.^11^

 The construction of healthy parenting is affected by insecurities and fears. The fragility of parenting implies that parents perceive themselves as less effective in their role, thus affecting parental self-efficacy (SE),^12^ that is, the belief or confidence that parents have in their ability to perform parenting tasks, make decisions and demonstrate emotions, motivation, cognition and response to child behavior.^13^ Parental SE may also be linked to feeding practices, such as breastfeeding and types of feeding offered.^14^ However, it has not yet been documented whether maternal SE can influence the growth of PI. Therefore, the objective was to identify maternal and neonatal factors associated with growth outcomes in PI born before 33 weeks of gestation, assessed at 24 months CA. 

## METHOD

 Prospective cohort study with PI less than 33 weeks born in the Western Paraná University Hospital, in the city of Cascavel, state of Paraná, from 2019 to 2021; discharged from the NICU and monitored at the institution’s high risk outpatient clinic up to 24 months CA. Infants of mothers using illicit drugs or psychiatric drugs, adolescents, PI who went for adoption, who had special health needs or morbidities and malformations that interfere with growth, use of gastrostomy, cardiac or gastrointestinal tract malformations, any genetic syndromes or who died during the monitoring period were excluded. 

 The study exposure variables were: maternal sociodemographic aspects, gestational morbidities, type of delivery, gestational age — GA (according to the best obstetric estimate), birth weight/GA ratio, maternal SE; neonatal variables: respiratory distress syndrome (RDS), sepsis, bronchopulmonary dysplasia (BPD), retinopathy of prematurity (ROP), intraventricular hemorrhage (IVH), all grades necrotizing enterocolitis (NEC), metabolic bone disease (MBD), use of total parenteral nutrition (TPN), weight loss during the first days (%), time to recovery of birth weight (days), time to achieve full enteral feeding (120 ml/kg/day) and days of NICU stay and post-discharge outcome, breastfeeding and anthropometric measurements at birth and discharge. 

 Follow up data were divided into periods, according to CA:

1. First month;
2. Two to three months;
3. Four to five months;
4. Six to eight months;
5. Nine to 12 months;
6. 13 to 18 months; and
7. 19 to 24 months.

Weight, height and head circumference (HC) Z-scores were calculated by the Fenton calculators from birth to 40 weeks and AnthroCalc, from the World Health Organization (WHO), after 40 weeks, both available online. AnthroCal provides z-scores for anthropometric indices, allowing comparison of child growth with WHO reference standards (2006/2007); CA was used for calculation in the present study. When there was more than one measurement in the periods, the Z scores were averaged. 

 To evaluate the SE for care (confidence that parents have in their ability to perform the tasks of parenthood), the Preterm Parenting and Self-efficacy Checklist^15^ questionnaire was made available to the mothers in periods V and VI of monitoring during consultations or via WhatsApp via Google Forms®. 

 This instrument presents 36 items, divided into three subscales, factors or domains (12 items each): 

 Factor 1: parental SE (beliefs and judgments that parents hold to organize and perform tasks related to the care of their children); 

 Factor 2: importance of tasks (how important parents feel to perform some tasks); 

 Factor 3: perceived parental competence (ability to perform certain tasks). 

 The instrument is self-administered and uses the seven-point Likert scale (1 – not at all confident; 2 – not confident; 3 – not very confident; 4 – insecure; 5 – somewhat confident; 6 – confident; 7 – very confident).^15^

 At the time of the first outpatient consultation, the mothers were informed about the study and invited to sign the Informed Consent Form. 

 Data were tabulated in an Excel spreadsheet, modeled and analyzed in specific statistical software: XSLStat Cloud 2020, New York, USA; Statistical Package for the Social Sciences, version 23, Armonk, USA; JASP, version 0.18.2.0, Amsterdam, the Netherlands. 

**Figure 1:**
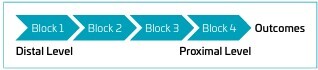
Block flowchart, according to clinical, temporal and logical-mathematical criteria of the variables that influence the growth of premature newborns under 33 weeks at 24 months of corrected age in Cascavel, state of Paraná.

 The first stage of data modeling aimed to check for possible typing errors, missing cases, extreme values and other factors that could compromise statistical modeling. Subsequently, descriptive statistics techniques were employed to operationalize the variables by blocks. The blocks were organized based on clinical, temporality (distal-proximal) and logical-mathematical criteria (Figure 1). 

 The statistical modeling process for the purpose of testing plausible causal predictive theories (Figure 1)^16^ can be understood as follows:

- Block 1 – Maternal demographic variables: age, education, occupation, income, marital status, number of children and pregnancies, previous experience with children and premature children. Gestational variables, according to obstetric records: intrauterine growth restriction (IUGR — fetus that fails to reach its growth percentage, caused by maternal and placental factors), clinical chorioamnionitis, diabetes mellitus, preeclampsia and use of antenatal steroids.
- Block 2 – Delivery variables: type of delivery (cesarean or vaginal), GA (according to the best obstetric estimate), birth weight/GA ratio (adequate [AGA] between the 10^th^ and 90^th^ percentile, small [SGA] below the 10^th^ percentile according to Fenton’s calculator).
- Block 3 – Neonatal variables: RDS, sepsis, BPD, ROP, IVH, all grades NEC, MBD, use of TPN, weight loss during the first days (%), time to recovery of birth weight (days), time to achieve full enteral feeding (120 ml/kg/day) and days of NICU stay.
- Block 4 – Need for hospitalization during the monitoring period of the study, breastfeeding duration and maternal SE.
- Outcome – Weight, height and HC at the end of the monitoring period (Period VI).

The study variables were inspected for bi- and multivariate normality by the Shapiro-Wilk test. Spearman’s or Pearson’s correlation analyses, Welch’s tests, χ2 or Fisher’s Exact Test were conducted. Welch’s test was adopted in comparisons of means when the size of the groups is unequal; association tests (χ2 or Fisher’s Exact Test) were used taking as an outcome the Z scores of the weight, height and HC at the end of the monitoring period and the respective categorization (success or failure in growth, defined as Z <-2 standard deviation (SD)).^17^ Associations with a p≤0.20 were included in regression, hierarchical linear block or logistic models^16^ to identify possible predictors of growth failure. 

 In the final linear prediction models, a p<0.05 was adopted. The outcome of the hierarchical regression analyses were the Z-scores of the anthropometric measurements and the final coefficients are expressed in betas (β) with respective 95% confidence intervals (CI). The requirements for conducting regression analyses were inspected, including multivariate normality, multicollinearity and heteroscedasticity.^18^ Kolmogorov-Smirnov tests were administered to ascertain whether the residuals of the models could potentially be attributed to a normal distribution. The results exceeded the established significance threshold for the final models (p>0.05). Variance inflation factors (VIFs) were employed to identify the presence of multicollinearity among predictors, with all calculated values remaining below 5.06. 

 The power of the study was calculated in the G*Power software (version 3.1). Thus, considering the hierarchical linear regression models conducted, number of independent variables inserted and taking the lowest variance explained, the power of the study was 91% (α <0.05); in the calculation, the effect size (f^2^) inserted was 0.26. 

 The study was approved by the Human Research Ethics Committee of the University of Western Paraná by Certificate of Presentation for Ethical Appreciation (CAAE) number 5.078.538. 

## RESULTS

**Figure 2:**
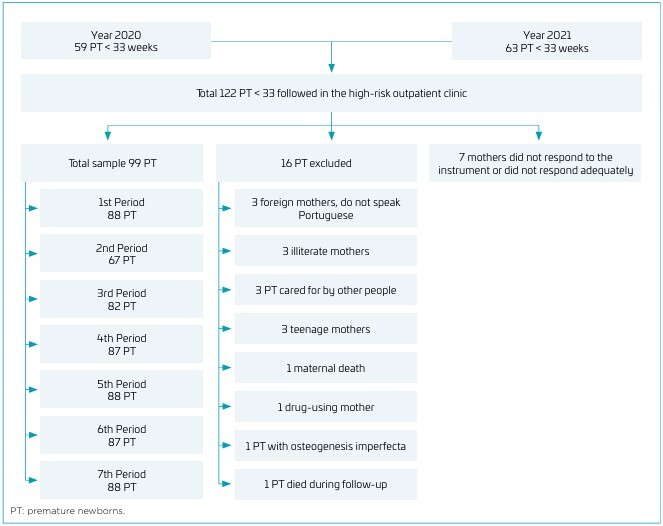
Flowchart of premature newborns under 33 weeks born in 2020 and 2021 followed up at the high-risk outpatient clinic up to two years corrected age, Cascavel, state of Paraná.

 Among 122 PI eligible for outpatient monitoring, 99 fulfilled the inclusion criteria, and 88 remained in the follow-up for 19–24 months CA (cohort loss 11%) (Figure 2). 

 The mean maternal age was 27.3 (±6.6) years, 68% of the mothers lived in a stable union, 52.7% had completed high school, 38.4% were housewives and 39.6% had a formal employment. The reported monthly income was up to two minimum wages (MW) for 40.7% of the families, and between two and four MW in 24%. Preeclampsia was the main gestational morbidity (25%) and cesarean section the main route of delivery (53.8%). 

 The mean GA of the cohort was 30.2 weeks (±2.0), 56.5% were male and 92% were AGA. Mean weight, height, and HC Z-scores at birth were -0.31 (±0.69), -0.4 (±0.86), and -1.16 (±0.87), respectively. RDS was the most frequent morbidity (81.1%). 

 Regarding nutritional data, 49 newborns received parenteral nutrition, and the time of use was 19±12 days. The mean weight loss in the first days was 11±5%. The PI took 13±5 days to regain birth weight, and required, on mean, 16 days to reach full enteral feeding (120 ml/kg/day). The mean hospital stay was 42±23 days and 76% were discharged on breastfeeding (exclusive or mixed). Regarding the SE scale, the average for domain 1 was 6.05, domain 2 was 6.15 and domain 3 was 6.23. 

 There was a significant, negative and moderate association between weight (p=-0.535, p=0.001), height (p=-0.342, p=0.001) and HC (p=-0.436, p=0.001) and time until reaching the full enteral feeding. Thus, the longer the time to reach the full enteral feeding, the lower the scores of the three anthropometric measures at two years CA. The time until reaching full enteral feeding explains 28% of the weight Z score in period VII of monitoring. A correlation was also identified for the length of stay in the NICU with the three anthropometric variables, and the length of stay explains 24% of the variable HC (Table 1). 

**Table 1:** Spearman correlations between continuous variables influencing the growth of premature newborns younger than 33 weeks at 24 months corrected age in Cascavel, state of Paraná.

		1	2	3	4	5	6	7	8	9	10	11	12	13	14	15
1. Weight (Period 7)	p	-														
	p	-													
2. Height (Period 7)	p	0.585 *	-													
	p	<0.001	-													
3. Head circumference (Period 7)	p	0.525 *	0.305 †	-												
	p	<0.001	0.004	-												
4. Self-efficacy - Factor 1	p	-0.276 †	-0.119	-0.270 ‡	-											
	p	0.009	0.268	0.012	-											
5. Self-efficacy - Factor 2	p	-0.106	-0.285 †	-0.077	0.646 *	-										
	P	0.324	0.007	0.480	<0.001	-										
6. Self-efficacy - Factor 3	p	-0.172	-0.052	-0.210	0.838 *	0.689 *	-									
	p	0.110	0.629	0.052	<0.001	<0.001	-									
7. Breastfeeding time	p	-0.047	-0.029	0.195	0.155	0.016	0.058	-								
	p	0.728	0.830	0.146	0.225	0.904	0.650	-								
8. Age of mother	p	-0.128	-0.018	-0.023	0.296 †	0.103	0.229 ‡	0.123	-							
	p	0.235	0.868	0.831	0.003	0.312	0.023	0.336	-							
9. Number of children	p	-0.164	-0.128	-0.182	0.320 †	0.179	0.244 ‡	0.173	0.419 *	-						
	p	0.128	0.233	0.094	0.001	0.076	0.015	0.176	<0.001	-						
10. Number of pregnancies	p	-0.230 ‡	-0.130	-0.202	0.314 †	0.234 ‡	0.246 ‡	0.226	0.476 *	0.807 *	-					
	p	0.031	0.226	0.062	0.002	0.020	0.014	0.075	<0.001	<0.001	-					
11. Gestational age	p	0.277 †	0.157	0.306 †	0.064	0.048	0.017	-0.008	-0.017	0.058	-0.017	-				
	p	0.009	0.145	0.004	0.529	0.634	0.870	0.953	0.867	0.571	0.869	-				
12. Time to reach full enteral feeding	p	-0.535 *	-0.342 †	-0.436 *	0.091	0.104	-0.145	-0.150	0.063	0.108	0.136	-0.608 *	-			
	p	<0.001	0.001	<0.001	0.370	0.306	0.153	0.240	0.534	0.289	0.179	<0.001	-			
13. Length of stay in NICU	p	-0.436 *	-0.315 †	-0.490 *	-0.032	-0.023	-0.020	-0.010	0.083	-0.008	0.080	-0.825 *	0.753 *	-		
	p	<0.001	0.003	<0.001	0.753	0.824	0.847	0.938	0.417	0.938	0.429	<0.001	<0.001	-		
14. % of weight loss	p	-0.141	-0.062	-0.007	-0.117	-0.028	-0.115	0.124	-0.046	0.004	-0.002	-0.123	0.142	0.038	-	
	p	0.194	0.569	0.949	0.252	0.785	0.263	0.338	0.653	0.969	0.983	0.231	0.166	0.713	-	
15. Time to regain birth weight	p	-0.087	0.038	0.047	-0.093	-0.044	-0.082	0.030	-0.085	-0.054	-0.041	-0.090	0.024	0.003	0.618	*
	p	0.425	0.730	0.671	0.366	0.667	0.425	0.817	0.410	0.599	0.691	0.383	0.816	0.981	<0.001	

Notes: *p<0.001; †p<0.01; ‡p<0.05.

Significant Shapiro-Wilk test for bivariate normality. Measures 1, 2 and 3 are expressed in Fenton’s Z-score.

 A negative and weak correlation was identified between maternal SE with weight and HC (p=-0.276, p=0.009; p=-0.270, p=0.012, respectively). Regarding the SE domains, the importance of tasks (Factor 2) correlated negatively and weakly with height (p=-0.285, p=0.007). It was observed that the higher the maternal age and the number of children, the higher the SE and parental competence (positive and weak correlation between maternal SE — Factor 1 and perceived parental competence — Factor 3). The three factors of the SE scale also showed a positive association with the number of pregnancies (p=-0.314, p=0.002; p=-0.234, p=0.020; p=-0.246, p=0.014, respectively). 

 The factors associated with Z scores and growth failure (Z score <-2) at 24 months CA were also explored. IUGR has a significant association with HC Z-score (p=0.006), with failure in weight (p=0.02) and height (p=0.01). Previous experience with children was associated with weight Z-score (p=0.01). Being born SGA was associated with Z-score of the three anthropometric measures (p=0.01 to <0.001) and growth failure for the three measures (p=0.003 to 0.01). Neonatal sepsis was associated (p<0.001) with the Z score of the three anthropometric measurements at the end of the monitoring period. BPD was associated with weight (p=0.03) and HC (p=0.006). The presence of ROP and the need for laser therapy influence the Z-score specifically of HC (p=0.03). Metabolic bone disease was associated with growth failure in height (p=0.04). The use of parenteral nutrition was associated with the Z-score of weight, height and HC (p<0.001). The other maternal and neonatal variables were not statistically significant. 

 The predictive logistic regression models include the variables that concomitantly presented associations with a p<0.20 in the bivariate analyses and that contained approximately ten observations per parameter to be estimated (Table 2). The accuracy of all models was greater than 90%. 

**Table 2:** Logistic regression with categorical predictors of growth failure in premature newborns younger than 33 weeks at 24 months of corrected age in Cascavel, state of Paraná.

Models		B	Error	χ^2^	p-value	OR	95%CI
Height*							
	No IUGR	-1.66	0.82	4.09	0.043	0.19	[0.04, .095]
	TPN	-1.59	1.13	1.98	0.160	0.20	[0.02, 1.87]
Weight†							
	SGA	2.70	0.85	10.15	0.001	14.83	[2.82, 77.93]
	RDS	-0.47	1.18	0.16	0.694	0.63	[0.06, 6.37]
HC‡							
	SGA	2.71	0.94	8.33	0.004	15.00	[2.39, 94.32]

OR: odds ratios; CI: confidence interval; IUGR: intrauterine growth retardation; TPN: total parenteral nutrition; SGA: small for gestational age; RDS: respiratory distress syndrome; HC: head circumference.

* χ^2^ (2)=8.88, p=0.012, Nagelkerke’s R2=21.04, Accuracy: 90%, AUC: 0.76;

† χ^2^(2)=9.76, p=0.008, Nagelkerke’s R2=21.31, Accuracy: 91%, AUC: 0.65;

‡ χ^2^(1)=7.51, p=0.006, Nagelkerke’s R2=21.05, Accuracy: 93%, AUC: 0.71. VIFs<1.50 on all models.

 Not having presented IUGR decreased approximately 81% the chances of growth failure in stature. Although the model retained the TPN variable, it is not possible to infer a direct effect on the height outcome, since the confidence interval contains the number 1. Overall, the logistic model explained about 20% of the variance. SGA increased the chance of failure in weight and HC growth by about 15 times at 24 months CA. 

 In the hierarchical linear regression analysis (Tables 3 and 4), considering the significant variables related to block four, having necrotizing enterocolitis decreased by 0.30 Z-score units for weight (β=-0.30, p=0.048). In addition, for each unit of maternal SE increase, there was a change of 0.37 in the height Z-score (β=-0.37, p=0.043) and, for each additional day to reach the full enteral feeding, there was a decrease of 0.41 in the HC Z-score (β=-0.41, p=0.045). The variables included in the model were number of pregnancies, previous experience with children and preterm children, IUGR, GA, SGA, time to achieve full enteral feeding, sepsis, BPD, ROP, laser therapy in severe ROP, days of TPN, need for readmission, maternal SE score and breastfeeding time (Tables 3 and 4). 

**Table 3:** Hierarchical linear regression according to anthropometric outcomes weight and height in premature newborns younger than 33 weeks at 24 months of corrected age in Cascavel, state of Paraná.

Models		B	Error	95%CI	β	t	p-value
Weight							
Block 1 (R^2^=0.31; F=3.66)*							
	Children	-0.13	0.22	[-0.57, 0.31]	-0.13	-0.60	0.552
	Preterm (no)	-0.001	0.41	[-0.84, 0.83]	-0.0005	-0.00	0.997
	IURG (no)	-0.16	0.46	[-1.10, 0.78]	-0.05	-0.35	0.725
	Pregnancies	0.07	0.21	[-0.36, 0.50]	0.08	0.32	0.747
	Experience with children (no)	-0.03	0.32	[-0.69, 0.62]	-0.01	-0.10	0.920
	Partner (yes)	-0.22	0.33	[-0.89, 0.44]	-0.09	-0.68	0.500
Block 2 (R^2^=0.40; ΔR^2^=0.09; F=2.27)†							
	Vaginal delivery	0.06	0.29	[-0.52, 0.64]	0.03	0.22	0.830
	Gestational age	-0.15	0.10	[-0.35, 0.06]	-0.26	-1.43	0.162
	Adequate for gestational age	-0.57	0.58	[-1.75, 0.61]	-0.14	-0.97	0.336
Block 3 (R^2^=0.57; ΔR^2^=0.17; F=3.15)*							
	Time to achieve full enteral feeding	-0.03	0.02	[-0.07, 0.008]	-0.35	-1.58	0.121
	Sepsis (no)	0.63	0.39	[-0.15, 1.42]	0.26	1.63	0.110
	Laser therapy (no)	0.20	0.68	[-1.19, 1.58]	0.04	0.29	0.775
	NEC (no)	-1.86	0.91	[-3.69, -0.02]	-0.30	-2.05	0.048
	TPN	0.79	0.43	[-0.08, 1.67]	0.34	1.84	0.074
Block 4 (R^2^=0.58; ΔR^2^=0.01; F=0.64)							
	Breastfeeding time	-0.01	0.02	[-0.05, 0.02]	-0.10	-0.81	0.421
	Self-efficacy	-0.006	0.007	[-0.02, 0.008]	-0.11	-0.83	0.413
Height							
Block 1 (R^2^= 0.19; F = 1.96)†							
	Pregnancies	0.09	0.24	[-0.41, 0.59]	0.10	0.37	0.714
	Preterm (no)	-0.03	0.52	[-1.09, 1.03]	-0.01	-0.07	0.949
	Children	-0.04	0.25	[-0.55, 0.48]	-0.04	-0.14	0.888
	Experience with children (no)	0.15	0.40	[-0.66, 0.96]	0.06	0.39	0.700
	IUGR (no)	0.42	0.54	[-0.68, 1.52]	0.13	0.78	0.441
Block 2 (R^2^=0.32; ΔR^2^=0.13; F=3.65)*							
	Gestational age	0.14	0.14	[-0.14, 0.43]	0.27	1.02	0.317
	SGA (no)	-1.36	0.68	[-2.75, 0.03]	-0.33	-2.00	0.054
Block 3 (R^2^=0.41; ΔR^2^=0.09; F=0.87)†							
	Time to achieve full enteral feeding	0.004	0.02	[-0.04, 0.04]	0.06	0.22	0.828
	Sepsis (no)	0.58	0.50	[-0.45, 1.61]	0.25	1.15	0.261
	BPD (no)	-0.71	0.61	[-1.97, 0.54]	-0.29	-1.16	0.256
	ROP (no)	-0.24	0.37	[-1.01, 0.52]	-0.10	-0.64	0.524
	Laser therapy (no)	0.24	0.78	[-1.34, 1.83]	0.05	0.31	0.756
	TPN	0.52	0.56	[-0.62, 1.67]	0.23	0.94	0.357
	Readmission (no)	0.34	0.46	[-0.59, 1.28]	0.12	0.75	0.462
Block 4 (R^2^=0.53; ΔR^2^=0.12; F=2.45)†							
	Self-efficacy	-0.02	0.009	[-0.04, -0.0007]	-0.37	-2.12	0.043
	Breastfeeding time	-0.02	0.02	[-0.06, 0.02]	-0.11	-0.77	0.445

CI: confidence interval; IUGR: intrauterine growth retardation; NEC: necrotizing enterocolitis; SGA: small for gestational age; BPD: bronchopulmonary dysplasia; ROP: retinopathy of prematurity; TPN: total parenteral nutrition; MW: minimum wage.

*p<0.05; †p≤0.10.

**Table 4:** Hierarchical linear regression according to anthropometric outcome (head circumference) in premature newborns younger than 33 weeks at 24 months of corrected age in Cascavel, state of Paraná.

Models		B	Error	95%CI	β	t	p-value
Head circumference							
Block 1 (R^2^=0.30; F=3.57)*							
	IUGR (no)	0.47	0.47	[-0.49, 1.43]	0.15	0.99	0.329
	Children	-0.41	0.21	[-0.82, 0.007]	-0.39	-1.99	0.054
	Income >2 MW	-0.41	0.34	[-1.09, 0.27]	-0.16	-1.22	0.228
	Experience with children	-0.46	0.36	[-1.19, 0.26]	-0.18	-1.29	0.204
	Pregnancies	-0.01	0.20	[-0.42, 0.39]	-0.02	-0.07	0.941
	Eclampsia (no)	0.16	0.42	[-0.69, 1.01]	0.06	0.38	0.710
Block 2 (R^2^=0.46; ΔR^2^=0.16; F=4.41)*							
	Vaginal delivery	-0.25	0.31	[-0.89, 0.38]	-0.11	-0.81	0.423
	SGA (no)	-1.09	0.62	[-2.34, 0.16]	-0.27	-1.76	0.086
	Gestational age	0.10	0.12	[-0.13, 0.34]	0.18	0.88	0.386
Block 3 (R^2^=0.54; ΔR^2^=0.08; F=1.21)							
	Time to achieve full enteral feeding	-0.03	0.02	[-0.07, -0.0008]	-0.41	-2.07	0.045
	Sepsis (no)	0.70	0.44	[-0.19, 1.59]	0.29	1.59	0.120
	BPD (no)	-0.49	0.53	[-1.56, 0.58]	-0.19	-0.93	0.357
	ROP (no)	-0.05	0.32	[-0.69, 0.60]	-0.02	-0.14	0.888
	Laser therapy (no)	-0.30	0.71	[-1.74, 1.13]	-0.06	-0.43	0.673
	TPN	-0.47	0.46	[-1.39, 0.46]	-0.20	-1.02	0.313
Block 4 (R^2^=0.57; ΔR^2^=0.03; F=1.16)							
	Self-efficacy	-0.01	0.007	[-0.03, 0.004]	-0.21	-1.46	0.154
	Breastfeeding time	0.006	0.02	[-0.03, 0.04]	0.05	0.38	0.705

*p<0.05.

IUGR: intrauterine growth retardation; MW: minimum wage; SGA: small for gestational age; BPD: bronchopulmonary dysplasia; ROP: retinopathy of prematurity; TPN: total parenteral nutrition.

## DISCUSSION

 Our data show that many factors related to neonatal period were associated with growth failure in the first years of life. Furthermore, it was observed that maternal sociodemographic and psychological factors influenced the preterm infant’s growth, highlighting the influence of maternal SE, an aspect that, to our best knowledge, has not yet been reported in literature. 

 In monitoring PI until hospital discharge, those with postnatal growth failure^19^ had longer TPN time and longer time to reach full enteral feeding. The longer the time to reach full enteral feeding, the lower were the scores of the three anthropometric measures at 24 months CA. Therefore, this growth deficit during hospitalization may persist in monitoring, reinforcing the importance of early initiation of enteral feeding with breast milk, preferably the mother’s milk, which impacts parenteral nutrition time, time to achieve full enteral feeding and length of hospital stay.^20,21^

 In very low birthweight infants (<1500 g), the incidence of growth failure during hospitalization is high, inversely related to GA and birth weight. Infants with moderate to severe BPD, IVH (high grade) or ROP demonstrate a slower growth velocity at 3–5 weeks postnatal, but these morbidities were not predictors of weight Z-scores at 36 weeks CA.^9^ In the present study, sepsis, BPD, MBD, severe ROP (need for laser) and time of use of TPN influenced the Z scores of anthropometric measurements at 24 months CA, but were not predictors of growth failure. On the other hand, NEC had a significant negative impact on weight growth in 24 months CA. 

 In a previous study conducted in the same region with a similar population, the presence of MBD and ROP negatively influenced the growth of PI, especially in the first trimester of life,^17^ highlighting the impact of morbidities associated with prematurity on postnatal growth.^7^

 The negative effect of IUGR, as well as being born SGA, on the growth trajectory of term and PI is well documented in the literature.^5,22,23^ In this study they were confirmed as predictors of growth failure in PI at 24 months CA. In a previous study of our group, PI born AGA were five times more likely to achieve a weight Z-score above -2.0 at 12 months CA.^17^

 In addition to the biological factors and morbidities widely discussed in the literature, psychosocial aspects can also impact the infant growth and should be investigated. Our results suggest that maternal SE can be a modulating factor of preterm infant growth in the first years of life. This deserves further investigation, as it is a new find, supported by the current family-centered focus of neonatal care.^24^ The NICU environment may compromise the growth of PI due to the stress they suffered, maternal depression and inadequate mother-child interaction.^25^ Since SE is closely related to emotional well-being in mothers of premature infants, increases in anxiety, perceived stress, and depression are associated with decreases in maternal SE.^26^

 As one of the sources of SE is the individual’s own previous experience,^3^ the present study identified that maternal SE and perceived parental competence had a positive correlation with maternal age, number of pregnancies and children. Studies have shown that multiparous women with less education have higher levels of SE,^27^ while mothers of premature infants with prolonged hospitalization in NICU have lower SE.^28^

 SE develops and increases as parents have more time and opportunities to practice and successfully complete parenting tasks. Despite the stress and anxiety of having a child in the NICU, parental SE increases as children grow and develop during hospitalization, highlighting the importance of parental participation in care.^29^

 The early identification of the health conditions of this most vulnerable group, beyond the biological and sociodemographic aspects, broadens the perception of the health-disease process of prematurity. Therefore, SE should be promoted since hospitalization, and the implementation of the Kangaroo Method is an important strategy to stimulate maternal SE and, consequently, healthy parenting.^30^

 While hierarchical models are highly effective for the examination of data characterized by inherent groupings, they necessitate meticulous examination of causal relationships as well as a clear distinction between fixed and random effects. Although the underlying assumptions of the model were rigorously evaluated, the findings must be interpreted within the contextual framework, acknowledging the limitations in establishing direct causality and the challenge of distinguishing fixed effects, which remain constant irrespective of the context, from random effects, which may fluctuate or be absent across varying contexts. 

 The limitations of this study are the inclusion of eight pairs of twins in the sample, which may influence the child’s growth and maternal SE, and the monitoring time that was limited to 24 months CA. However, the cohort loss was only 11%, so it was possible to achieve the objective with the sample studied and the results brought new information. The identification of clinical and psychosocial factors that influence early growth contributes to guide and improve neonatal care practices, stimulating further research to better understand the role of maternal SE in the growth prognosis of PI. 

 In conclusion, the growth of PI in the first 24 months CA is influenced by nutritional condition at birth, nutritional evolution during hospitalization and maternal SE. Optimizing nutritional practices in NICU and stimulating maternal SE are possibilities to improve growth of PI in the first years. 

## Data Availability

The database that originated the article is available with the corresponding author.
